# TCR and BCR repertoire analysis reveals distinct signatures between benign and malignant ovarian tumors

**DOI:** 10.3389/fonc.2025.1630707

**Published:** 2025-08-11

**Authors:** Zhonghuang Wang, Zhe Zhang, Dongli Zhao, Zhenglin Du, Bixia Tang, Enhui Jin, Hailong Kang, Wenming Zhao, Yuanguang Meng

**Affiliations:** ^1^ National Genomics Data Center, Beijing Institute of Genomics, Chinese Academy of Sciences/China National Center for Bioinformation, Beijing, China; ^2^ Department of Obstetrics and Gynecology, Seventh Medical Center of Chinese PLA General Hospital, Beijing, China; ^3^ Changping Laboratory, Beijing, China; ^4^ Gynecological Mini-Invasive Center, Beijing Obstetrics and Gynecology Hospital, Capital Medical University, Beijing Maternal and Child Health Care Hospital, Beijing, China

**Keywords:** ovarian tumors, TCR, BCR, machine learning, biomarks

## Abstract

**Background:**

The immune system is of paramount importance in maintaining human health and defending against pathogens. Among them, the adaptive immune system is a crucial component of the immune system, as it is responsible for generating and modulating the immune repertoire, which is vital for immune responses.

**Methods:**

We conducted a comprehensive analysis of T cell receptor (TCR) and B cell receptor (BCR) clonotypes in the peripheral blood immune repertoire of 20 patients with benign and malignant ovarian tumors. The analysis elucidates the differences between the two immune repertoires in various aspects and constructs an early screening machine learning model for ovarian tumors based on the characteristics of the immune repertoire.

**Result:**

The finding revealed that patients with malignant ovarian tumors exhibited a reduction in balance, richness, and diversity in their immune repertoires compared to those with benign tumors. Additionally, there was a negative correlation between patient age and immune repertoire diversity, and the immune repertoire of patients with malignant tumors displayed high heterogeneity. By employing machine learning techniques, we have developed an early screening model based on 16 TCR V-J genes and 11 BCR V-J genes, which achieved an average AUC of 0.93 (TCR) and 0.958 (BCR) on the ovarian tumor test dataset. Moreover, a comparison of the spatial distributions of TCR and BCR revealed, for the first time, that TCR was more significantly associated with the benign-to-malignant transformation of ovarian tumors.

**Conclusions:**

Our study highlights the critical role of the adaptive immune repertoire in distinguishing between benign and malignant ovarian tumors. TCR demonstrated more distinct spatial distribution patterns between benign and malignant states, suggesting its potential as a more sensitive biomarker for ovarian tumor detection. These findings provide new insights into the immunological landscape of ovarian tumors and offer a promising avenue for early diagnosis and prognosis assessment.

## Introduction

Ovarian tumors, which have their origin in the ovaries, represent one of the most prevalent forms of neoplasia within the female genital tract Ovarian cancer (OC) represents a significant global health challenge, with a ranking as the eighth leading cause of cancer-related death in women. Furthermore, it is one of the most refractory malignancies in gynecology, with a 5-year survival rate of only 47% (1). Globally in 2022, ovarian cancer accounted for over 324,398 newly reported cases and nearly 206,839 deaths (2). Early-stage ovarian cancer is often asymptomatic or presents with non-specific symptoms, making early detection difficult, which complicates early detection. Consequently, many patients are diagnosed only once the cancer has spread beyond the ovaries. Current treatment options for ovarian cancer (OC) include platinum-based chemotherapy following primary surgery, or interval debulking surgery after neoadjuvant chemotherapy, followed by continued chemotherapy. However, the efficacy of these treatments is limited to a specific subset of patients, and the overall prognosis for ovarian cancer remains disconcerting (3). The most effective strategy for improving survival rates is early diagnosis. Studies reveals that approximately 93% of patients diagnosed at an early stage, such as stage IA or IB, with a small tumor size or local disease, have a greater likelihood of surviving for more than five years (4). At present, screening methods that combine the assessment of cancer antigen 125 (CA125) levels through blood tests with transvaginal ultrasound are particularly effective for the detection of OC. Nevertheless, the impact of widespread screening on overall survival rates remains to be substantiated. Despite the availability of basic screening methods for high-risk populations, the question of their efficacy in reducing mortality remains a significant challenge. Consequently, there is an increasing requirement for more specific biomarkers to improve early detection (5).

In the immune response, specific T cells are clonally expanded after activation to differentiate into helper T cells or cytotoxic subsets that recognize infected histiocytes by antigens presented by HLA-II or HLA-I molecules. Since the T cell response depends on antigen recognition, the type of tumor antigen presented by cancer cells affects the TCR repertoires. Tumor antigens are typically classified as either tumor-associated antigens (TAA) or tumor-specific antigens (TSA). TAA expression is highly elevated in tumor cells, whereas TSA expression is restricted to cancer cells and absent in healthy cells. Both TAA and TSA induce anti-tumor responses and promote the expansion of specific T cells. The highly variable complementarity-determining region 3 (CDR3) of the TCRβ chain is unique to each T cell clone, making monitoring the TCR immune repertoire a powerful tool for observing disease dynamics. Several studies have confirmed that the diversity and similarity levels of TCR sequences between tumor tissues and normal tissues can provide valuable insight into patient prognosis, with lower diversity associated with poorer prognosis (6–9). A high diversity of the TCR repertoires may be associated with better health status and better outcomes. Overall, healthy individuals with greater TCR diversity, as well as patients with more favorable disease progression, tend to have better immune system function and are more capable of mounting effective anti-tumor responses.

TCR diversity signatures are detectable not only in tissues but also in peripheral blood, enabling liquid biopsy approaches for clinical monitoring. For instance, an investigation of TCR diversity in the peripheral blood of patients with stage I-IV melanoma revealed a correlation between higher diversity and improved survival, while lower diversity is associated with a poorer prognosis (10). A similar association was observed in a study of breast cancer patients with lymphopenia and reduced TCR diversity in the peripheral blood, as measured by the lymphatic-diversity reduction index. These patients exhibited a significantly higher risk of early mortality (11). These consistent findings across cancer types validate peripheral blood TCR repertoire analysis as a clinically informative and minimally invasive monitoring tool.

Despite the ambiguity surrounding the underlying causes of conserved changes in TCR rearrangements across patients with different genetic backgrounds remain unclear, the concept of immunoediting (12) provides a framework for understanding the early signals observed. Specifically, early exposure to tumor antigens may trigger the rapid expansion of cancer-associated T cells (13), resulting in detectable signals of TCR rearrangements in circulating leukocytes. However, few studies have explored the discrepancies and nuances between early and late tumor immune repertoire TCRs/BCRs in peripheral blood. To address this gap, we conducted a study analyzing preoperative blood samples from ovarian tumor patients. We compared immune repertoire characteristics and enriched TCR/BCR biomarkers between individuals with benign ovarian tumors and those with malignant ovarian cancer. Our objectives were threefold: (1) to map the spatial distribution of clonotypes and gene families in these cohorts, (2) to evaluate how the phenotypic features of key TCRs/BCRs influence immune responses in benign versus malignant tumors, and (3) to clarify their roles in shaping the tumor microenvironment. By elucidating these mechanisms, we aim to leverage immune repertoire signatures to develop early-warning tumor detection models. Such models hold promise for advancing early clinical screening strategies and improving diagnostic outcomes.

## Materials and methods

### Patients

This study investigated the diversity of TCR (T-cell receptor) and BCR (B-cell receptor) immune repertoire characteristics in peripheral blood of patients with ovarian tumors, comparing benign and malignant subtypes. A total of 20 female patients (aged 16–69 years) were recruited preoperatively at the General Hospital of the Chinese People’s Liberation Army between 2020 and 2022. The cohort included 12 patients with benign ovarian tumors (ovarian teratomas, mucinous cystadenomas, and ovarian endometriosis cysts) and 8 patients with malignant ovarian tumors (high-grade serous carcinoma, serous cystadenocarcinoma, and low-grade adenocarcinoma). Preoperative peripheral blood mononuclear cells (PBMCs) were collected in EDTA-containing tubes, aliquoted, and stored at -80°C for subsequent TCR/BCR high-throughput sequencing. Tumor diagnoses were confirmed by histopathological review performed independently by two senior pathologists. The study adhered to the tenets of the Declaration of Helsinki and was approved by the Ethics Committee of the General Hospital of the Chinese People’s Liberation Army (approval no. S2022-403). Written informed consent was obtained from all participants prior to enrollment. Detailed clinical and demographic characteristics of the cohort are summarized in **Table 1**.

**Table 1:** Detail of patients.

No.	Sample	Age	Diagnose	Gravida	Para	Abortus	Group
1	Malignant_1	68	High grade serous ovarian cancer	4	2	2	Malignant
2	Malignant_2	48	High grade serous ovarian cancer	3	2	1	Malignant
3	Malignant_3	58	High grade serous ovarian cancer	2	1	1	Malignant
4	Malignant_4	69	High grade serous ovarian cancer	3	3	0	Malignant
5	Malignant_5	59	Poorly differentiated adenocarcinoma of ovary	1	1	0	Malignant
6	Malignant_6	52	High grade serous ovarian cancer	2	1	1	Malignant
7	Malignant_7	69	High grade serous ovarian cancer	4	3	1	Malignant
8	Malignant_8	58	High grade serous ovarian cancer	2	2	0	Malignant
9	Benign_1	60	Ovarian endometriosis cyst	1	1	0	Benign
10	Benign_2	51	Ovarian cystadenoma	2	2	0	Benign
11	Benign_3	44	Ovarian endometriosis cyst	1	1	0	Benign
12	Benign_4	48	Ovarian Teratoma	2	2	0	Benign
13	Benign_5	36	Ovarian endometriosis cyst	1	0	1	Benign
14	Benign_6	16	Ovarian Teratoma	0	0	0	Benign
15	Benign_7	55	Mucinous cystadenoma	2	1	1	Benign
16	Benign_8	32	Ovarian Teratoma	0	0	0	Benign
17	Benign_9	57	Mucinous cystadenoma	5	1	4	Benign
18	Benign_10	27	Ovarian Teratoma	1	1	0	Benign
19	Benign_11	41	Ovarian endometriosis cyst	3	2	1	Benign
20	Benign_12	30	Ovarian Teratoma	0	0	0	Benign

### TCR and BCR high-throughput sequencing

The high-throughput RNA multiplex sequencing workflow for BCR/TCR analysis included several key steps. Initially, peripheral blood mononuclear cells (PBMCs) were isolated from whole blood using Ficoll density gradient centrifugation, ensuring cell viability was ≥80% and a minimum cell count of 1×10^6. Total RNA was then extracted employing the Trizol method. The quality of the extracted RNA was assessed by Qubit 3.0 fluorometer (with a requirement of total RNA ≥2 μg) and NanoDrop2000 spectrophotometer (with an OD260/280 ratio maintained between 1.8 and 2.1). The integrity of the RNA was further confirmed by agarose gel electrophoresis, which was used to check for clear bands and ensure a 28S/18S rRNA ratio of ≥1:1. Subsequently, BCR-H sequencing libraries were constructed through multiplex PCR amplification, which targeted the CDR3 region of the BCR and TCR chains and covered all functional IGHV genes and TCR V/J rearrangements. The quality control of the library involved quantifying the DNA concentration (≥2 ng/μL) using Qubit 3.0 and confirming the fragment size (~300 bp) by agarose gel electrophoresis to ensure there was no primer-dimer contamination. The libraries were then pooled based on their concentration and tagged for identification, with a minimum concentration of 5 ng/μL. Finally, sequencing was carried out on an Illumina platform using a PE150 bp strategy, with a target of ≥6M PE reads per sample and a Q20 value of ≥90%. The Sequencing-By-Synthesis (SBS) principle was followed during sequencing, which involved amplifying the fragments into clusters and performing high-throughput parallel sequencing with fluorescently labeled reversible terminators.

### Identification and preprocessing of sequencing data for TCR/BCR

The raw sequencing data were processed through a standardized preprocessing pipeline to generate high-quality clean data. First, sequence quality was assessed using FastQC (14), with reads were then quality-filtered by trimming bases with Phred scores <20 and discarding reads with either <90% of bases having Phred scores ≥30 or post-trimming lengths <50 bp. Data quality was validated before proceeding to downstream immune receptor.

The resulting clean data were then analyzed using MiXCR (15) software, which identifies TCR and BCR clonotypes by aligning sequencing data to IMGT database reference gene fragments (V, D, J, C). After analysis, reads containing adapter sequences (>5% adapter contamination) or undetermined bases (N >5%) were removed, and only samples with successfully aligned reads >85% were retained; otherwise, samples were subjected to re-sequencing. MiXCR generates quantitative clonotype data by grouping sequences with shared characteristics, allowing flexible assembly criteria (e.g., focusing on CDR3 or specific VDJ regions). Its error-correction process includes mass correction for sequencing errors and low-quality reads, as well as PCR error correction via clustering and fuzzy matching to differentiate true clonotypes from artifacts.

To further refine the data, VDJtools (16) was employed for frequency-based correction to eliminate mismatched clonal pairs and normalize smaller clones to the dominant clone through three sequential steps: (1) Correct module merged erroneous clonotypes differing by ≤2 nucleotides when their abundance ratio was <0.05(mismatches), with correction restricted to clonotypes sharing identical V/J gene assignments; (2) Decontaminate module removed cross-sample contaminants by filtering clonotypes showing ≥20-fold higher abundance in other samples; (3) FilterNonFunction module excludes non-functional clones (e.g. those with stop codons or frameshift mutations), as these do not contribute to immune function. (4) DownSample module normalized sequencing depth by randomly subsampling to a minimum number of reads per sample using a fixed random seed.

### Statistical analysis

In the analysis of clonal diversity within a community, the Gini coefficient, the Chao1 index, and the Inverse Simpson Index are used to assess the distribution, richness, and diversity of clones. Each of these indices offers unique insights into different aspects of clonal distribution and composition. (1) The Gini coefficient measures the inequality of clone distribution in a sample, with values ranging from 0 to 1. A Gini coefficient closer to 0 indicates a more uniform distribution of clones, while a value closer to 1 suggests greater clonal variation, where a few clones dominate. A lower Gini coefficient reflects more evenness in clonal distribution. The formula for calculating the Gini coefficient is:


$$Gini=\frac{\sum_{i=1}^{n}\sum_{j=1}^{n}\left|x_{i}-x_{j}\right|}{2n^{2}\bar{x}}$$

Where n is the number of clones, $x_{i}$ is the relative abundance of the i−th clone, and $\bar{x}$ is the average relative abundance.

(2) The Chao1 index is used to estimate the richness of a community, considering not only the clones observed in the sample but also those likely unobserved. It accounts for clones that appear only once or twice, offering a more accurate reflection of the true species richness. A higher Chao1 index suggests greater richness in unobserved clones. The formula is:


$$S_{\text{chao}1}=S_{obs}+\frac{a_{1}(a_{1}-1)}{2(a+1)}$$

Where S_Chao1_ is the number of observed clones, a_1_ is the number of clones occurring only once, a_2_ is the number of clones occurring twice.

(3) The diversity index reflects the overall state of species richness and evenness, and the Inverse Simpson Index is a commonly used index, with values typically ranging from 1 to the number of species, with closer to 1 indicating lower diversity and higher indexes indicating higher diversity. The calculation formula is as follows:


$$Inv-Simp=\frac{1}{\sum_{i=1}^{S}p_{i}^{2}}$$

where Inv-Simp represents the inverse Simpson index, and S represents the number of species in the sample, representing the relative abundance of the i species in the sample.

The computational and statistical analysis of this study was performed using the R programming language (v4.3.2) and python (3.9.6), combined with the R package Immunarch for downstream data analysis and processing. The equilibrium of the immune repertoire was assessed by calculating the Gini Index, Chao1, and Inverse Simpson (Inv-Simp) indices. The spatial distribution of the immune repertoire was analyzed using the diversity module in the Immunarch package in R. For data visualization, the R software package ggplot2 was used to plot the violin plot and the correlation scatter plot. The calculation of differential clonotypes was performed using the limma-voom software package, combined with the ggplot2 and pheatmap packages in the R software, to create the volcano map and heat map display, respectively. The development of the early screening model was facilitated by a machine learning-based approach, implemented through the utilization of the R software package workflowsets.

### Machine learning model for early screening of ovarian tumors

To establish a robust biomarker model for the early diagnosis of benign and malignant ovarian tumors using peripheral blood, the following procedures were conducted: (1) Data preprocessing: V-J gene data in peripheral blood samples were cleaned by removing outliers and missing values. The frequency data of V-J genes were standardized to ensure consistent scaling across different genes. (2) Feature selection: 16 pairs of V-J genes with significant differences were screened as input for training traits, while 11 pairs of V-J genes with significant differences were selected as input for training features. (3) Data division: The dataset stratified random splitting using initial_split (prop = 0.7, strata = group) to partition the dataset into training (70%) and testing (30%) sets, preserving the original class distribution (60% benign, 40% malignant). The final subsets comprised: training set (14 samples: 8 benign, 6 malignant) and test set (6 samples: 4 benign, 2 malignant), ensuring proportional representation of both classes. (4) Model construction & validation: Three machine learning algorithms (Support Vector Machine, Random Forest, and Logistic Regression) were implemented using default hyperparameters to enable unbiased baseline performance comparison. To ensure rigorous evaluation, we employed stratified 3-fold cross-validation (vfold_cv(v = 3, strata = group)), training on 2 folds and validating on the third, with class ratios maintained in each fold. Performance metrics were aggregated across all repetitions. (5) Evaluation: Model efficacy was quantified primarily through ROC-AUC, which comprehensively assesses discriminative ability across all classification thresholds. This metric provides a reliable assessment for ovarian tumor screening, as it evaluates the model’s capacity to distinguish benign from malignant cases.

## Results

### Clonal statistical analysis in immune repertoire

**Figure 1:**
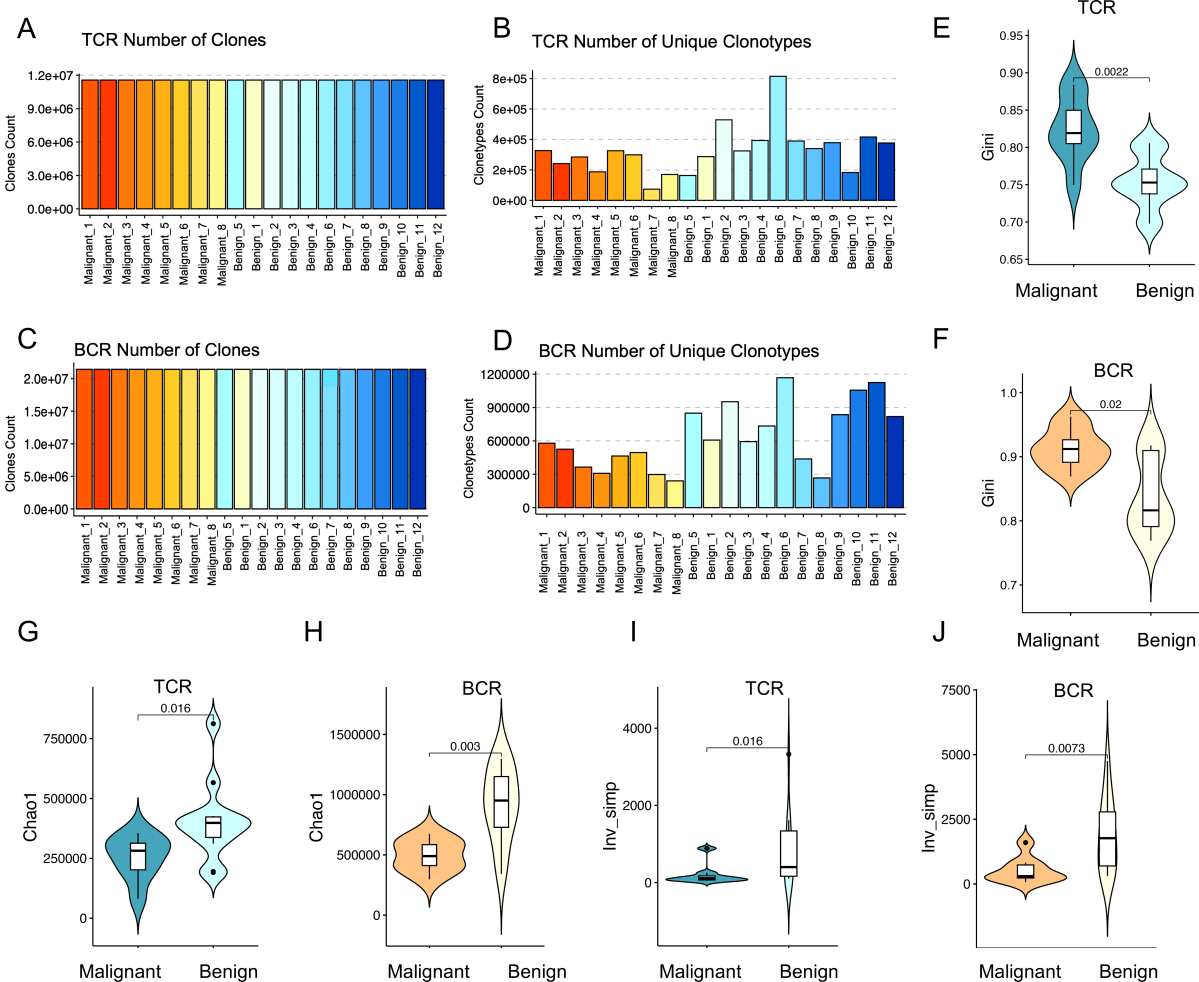
Diversity of immune repertoires among patients with benign and malignant ovarian tumors. **(A, C)** Clone counting with standard normalization for TCR and BCR samples, respectively. All samples were down-sampled to 11,503,414 clones for TCR and 23,546,466 clones for BCR to ensure normalization. Red bars indicate malignant tumor patients (n=8) and blue bars represent benign tumor patients (n=12). **(B, D)** The number of unique clonotypes for TCR and BCR, respectively. The horizontal axes of the bar plots represent individual sample names, with the first 8 samples corresponding to ovarian malignant patients and the last 12 samples representing ovarian benign patients. **(E, F)** Violin plots showing the differences in Gini coefficients for TCR (p=0.0055) and BCR (p=0.082) immune repertoires between malignant (dark color) and benign (light color) ovarian tumor patients. **(G, H)** Violin plots illustrating the differences in the Chao1 index for TCR (p=0.069) and BCR (p=0.02) immune repertoires between malignant (dark color) and benign (light color) ovarian tumor patients. **(I, J)** Violin plots presenting the differences in the inverse Simpson index for TCR (p=0.02) and BCR (p=0.025) immune repertoires between malignant (dark color) and benign (light color) ovarian tumor patients. Statistical comparisons were performed using the Wilcoxon-Mann-Whitney test.

The number of sequencing reads obtained from TCR receptor panels across a cohort of 20 patients was 11,503,414 - 24,988,362. Clones were identified in each sample using MiXCR, and clone counts were normalized by downsampling all samples to the level of the sample with the lowest clone count, which was 11,503,414 clones (**Figure 1A**). This normalization process helps account for errors and reduces file size for faster computations. After normalization, a range of 73,488 – 815,062 unique clone types per sample were identified (**Figure 1B**), representing unique clones (V+CDR3+J). Clones included 624 V-J panels, 48 different V gene segments, and 13 different J gene segments. For BCR sequencing, the mean number of reads per BCR immune panel was 23,546,466 - 43,039,304. Subsequent to clone identification, all samples were downsampled to 23,546,466 clones (**Figure 1C**). The analysis yielded a total of 267,338 – 1,167,848 distinct CDR3 amino acid clonotypes, encompassing 348 V-J gene panels, 58 distinct V gene fragments, and 6 different J gene segments (**Figure 1D**).

The number of unique clonotypes serves as a crucial indicator of immune repertoire diversity. A higher TCR clonotype count correlates with an enhanced capacity for antigen recognition, reflecting a more robust and diverse adaptive immune response. As depicted in **Figure 1B**, the eight ovarian malignancy samples (represented by red bars, with different shades indicating individual samples) exhibited a significantly reduced number of unique clonotypes compared to the 12 benign ovarian tumor samples (represented by blue bars, with different shades indicating individual samples). This observation was further supported by statistical analysis using the Wilcoxon rank-sum test, which revealed significant differences between malignant and benign samples, with p-values of 0.02 for TCR and 0.003 for BCR (**Supplementary Figure S1**). These p-values provide strong evidence that malignant samples have significantly fewer unique clonotypes compared to benign ones. This finding suggests a diminished immune repertoire richness in ovarian cancer patients relative to individuals with benign ovarian tumors. Similarly, BCR clonotype diversity was notably lower in the majority of ovarian malignancy patients compared to those with benign tumors, further indicating a compromised BCR immune repertoire in ovarian cancer.

### Lower immune repertoire diversity in malignant compared to benign tumors

In order to assess the differences in the peripheral blood immune repertoire between patients with malignant and benign ovarian tumors, three key indices were employed: evenness, richness, and diversity. (1) The evenness of the immune repertoire was quantified using the Gini index, which measures the degree of clonal imbalance, with higher values indicating increased dominance of a few expanded clones. As demonstrated in **Figures 1E, F**, the TCR and BCR repertoires in patients with malignant ovarian tumors exhibited significantly greater heterogeneity compared to those in patients with benign ovarian tumors (TCR: p = 0.0022; BCR: p = 0.02; Wilcoxon tests). This finding indicates that malignancy is associated with an imbalanced immune repertoire, characterized by the overexpansion of specific clones that disrupts the equilibrium of the immune repertoire. (2) The richness of the immune repertoire was evaluated using the Chao1 index, with higher values indicating a greater unobserved clone richness within the sample. As illustrated in **Figures 1G, H**, a significant disparity in the Chao1 index was observed between TCR and BCR in the two groups, indicating that the immune repertoire richness in patients with malignant ovarian tumors was considerably diminished in comparison to patients with benign ovarian tumors (TCR: p=0.016; BCR: p=0.003; Wilcoxon tests). This reduction in richness is likely attributable to the clonal expansion of specific clones. The diversity of the immune repertoire was assessed using the inverse Simpson index, which quantifies both richness and evenness. Higher values indicate greater diversity and a more even distribution of clonotypes, whereas lower values suggest a skewed repertoire dominated by specific clonotypes. As illustrated in **Figures 1I, J**, a significant decrease in peripheral blood immune repertoire diversity was observed in malignant ovarian tumor patients compared to those with benign tumors (TCR: p = 0.016; BCR: p = 0.0073; Wilcoxon tests). This suggests that as tumor malignancy increases, the diversity of the immune repertoire decreases, which may be correlated with a lower level of immune protection.

The findings revealed that the diversity of the peripheral blood immune repertoire, as measured by both TCR and BCR, was significantly reduced in patients with malignant ovarian tumors compared to those with benign ovarian tumors. This suggests that as tumor malignancy increases, the diversity of the immune repertoire also decreases, which may correlate with a lower level of immune protection. Enhancing immune repertoire diversity may thus represent a promising avenue for therapeutic interventions in oncological diseases.

### Increasing age correlates with decreased diversity in the immune repertoire

**Figure 2:**
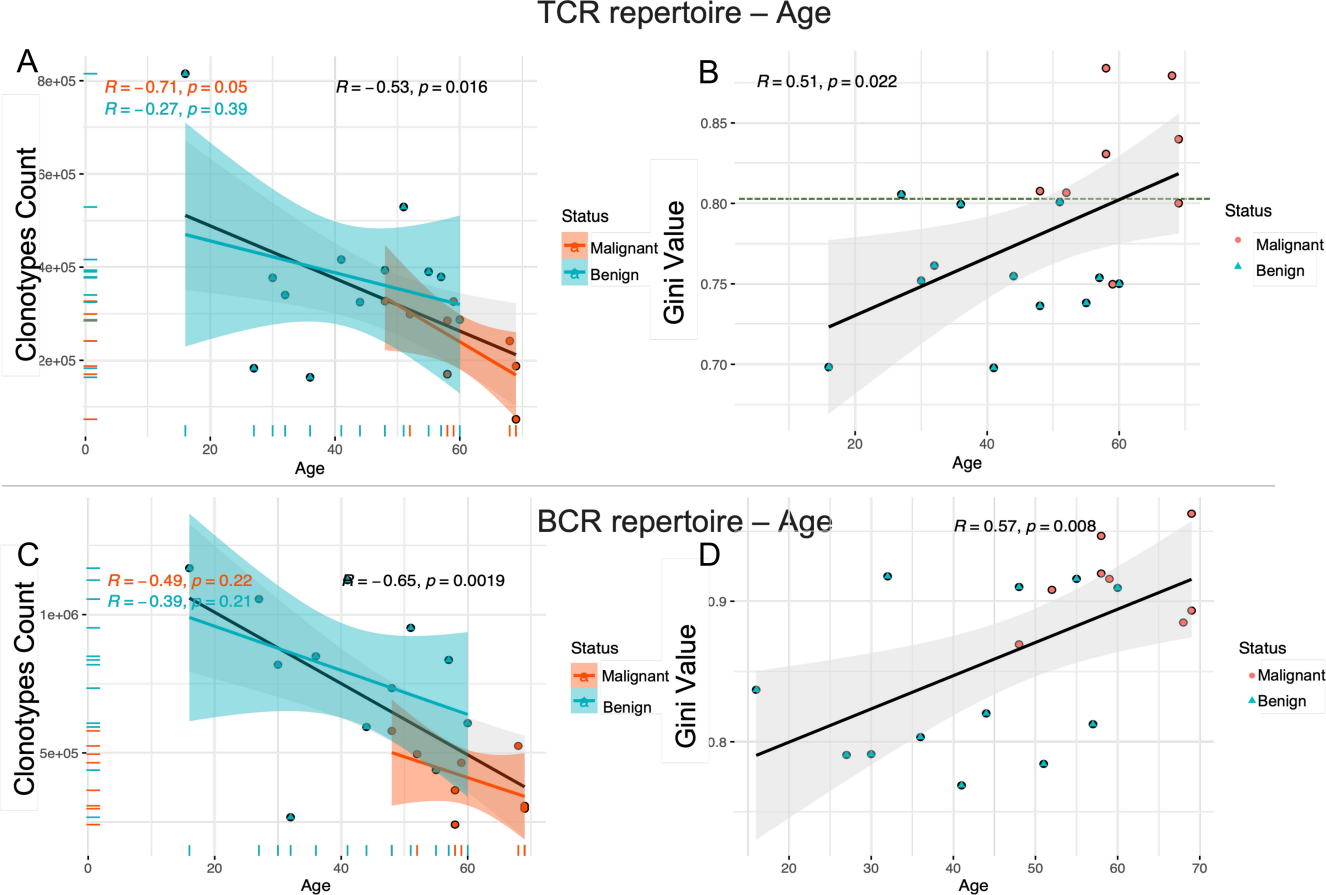
The decrease in immune repertoire diversity was associated with age. Scatter plots **(A, C)** show the correlation between the number of unique clonotypes and age for the TCR and BCR immune repertoires. Scatter plots **(B, D)** show the relationship between Gini coefficients and age for the TCR and BCR immune repertoires. The strength and direction of the linear relationship between these variables are quantified using the Pearson correlation coefficient (PCC). The blue line represents the fitted curve for ovarian benign patients, the red line corresponds to malignant patients, and the gray line shows the overall fitted curve for all samples. The horizontal axis represents age and the vertical axis represents the number of unique clonotypes or Gini index. The correlation coefficient (r) and the significance level (p) of the correlation are provided for each group.

As individuals age, the immune system undergoes a gradual decline, leading to reduced immune cell function, a decrease in cell numbers, and a weakened immune response. This aging process may also impact the diversity of the β-repertoire of T cells (TCR) and the H-chain repertoire of B cells (BCR). To assess whether the diversity of these immune repertoires decreases with age, we analyzed the relationship between the number of unique clonotypes and age in the TCR immune repertoire. The results shown in **Figure 2A** demonstrate a significant negative correlation between TCR diversity and age. Specifically, TCR diversity exhibited a marked decline with age in malignant patients (R = -0.71, p = 0.05), while a more modest decrease was observed in benign patients (R = -0.27, p = 0.39), and a moderate decrease in TCR diversity was evident in the overall cohort (R = -0.53, p = 0.0016). The age distribution revealed that malignant patients were generally older than benign patients (mean age > 50 years). At the same time, the Gini coefficient was positively correlated with age (**Figure 2B**), and the higher the Gini coefficient, the more obvious the TCR immune repertoire imbalance, which increased with age (R=0.51, p=0.022). Notably, a Gini coefficient of 0.8 may serve as a potential cut-off point to discriminate between benign and malignant tumors, although further validation with a larger sample size is needed. A similar pattern of age-related decrease in diversity was observed in the BCR immune repertoire (R = -0.65, p = 0.0019) (**Figure 2C**). Additionally, the Gini coefficient, which measures the inequality of clonotype distribution, was positively correlated with age (R = 0.57, p = 0.008) (**Figure 2D**) suggesting that both the TCR and BCR repertoires exhibit decreased diversity with increasing age.

Meanwhile, we investigated potential associations between reproductive history and immune repertoire. The analysis revealed positive trends for gravida, para, abortus with TCR diversity (Gini coefficients), though abortus-BCR showed negative trends, suggesting that increased reproductive events may correlate with clonal expansion (reduced diversity). However, these correlations did not reach statistical significance (p > 0.05 for all) in our cohort of 20 participants. This may be due to limited statistical power caused by the small sample size (see **Supplementary Figures S2**, **S3**).

The findings indicate that aging is associated with a progressive decline in the diversity of the immune repertoire in both T cells and B cells, accompanied by an increase in immune imbalance. This deterioration in immune equilibrium may contribute to a diminished capacity of the immune system to effectively eliminate tumor cells, thereby heightening the risk of tumor initiation and progression. Consequently, aging can be considered a significant contributing factor to tumorigenesis and disease advancement.

### Higher clonal heterogeneity in malignant ovarian tumors compared to benign

**Figure 3:**
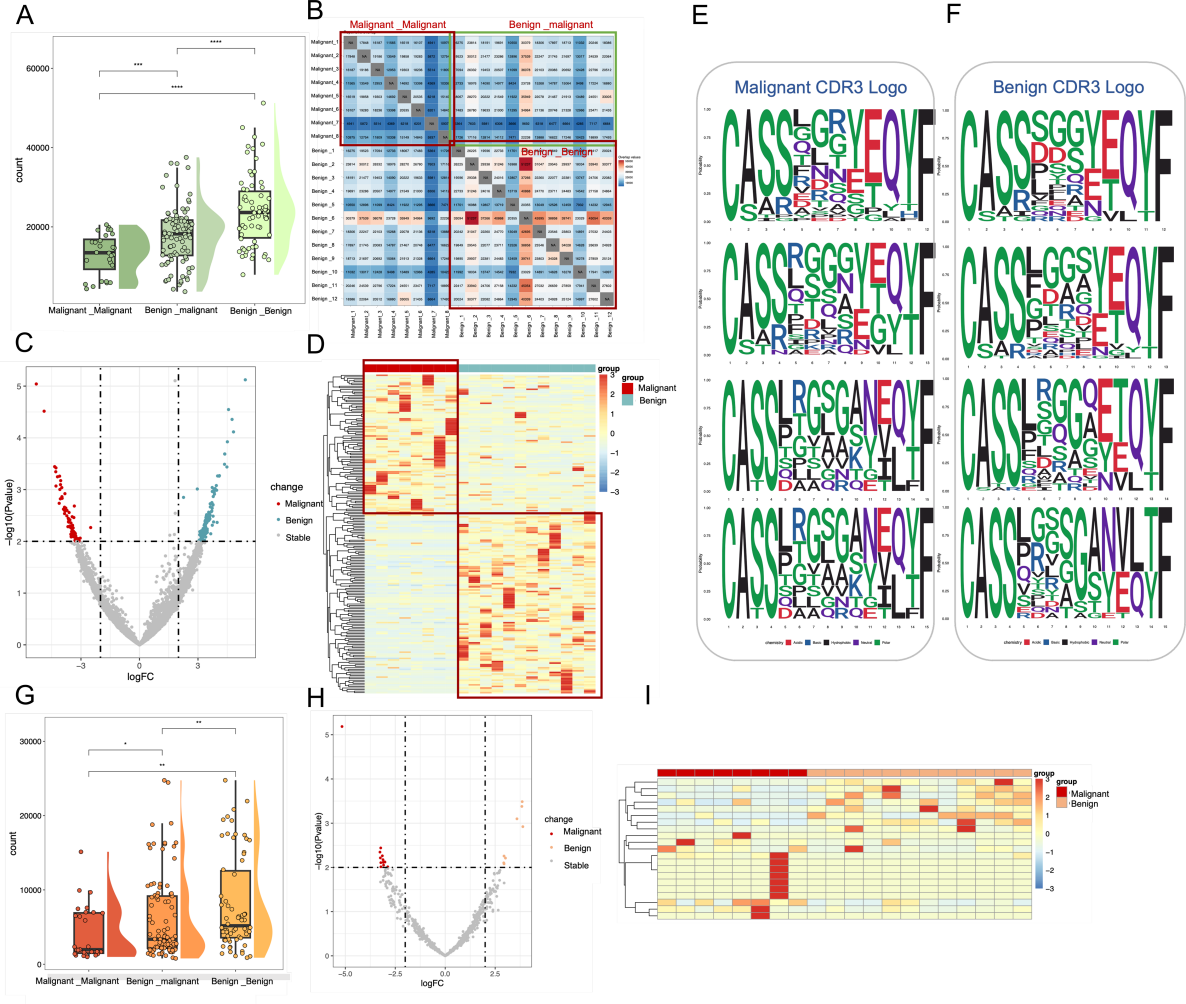
Characterization of shared Clone CDR3 between benign and malignant groups in TCR and BCR. **(A)** Box line plots demonstrating the differences in the number of shared clonal CDR3 in TCR immune repertoires among three groups: between malignant patients, between malignant and benign patients, and between benign patients. These correspond to the three red boxes in the TCR heatmap. **(B)** where a redder color indicates a higher number of shared CDR3. **(C)** Volcano plots based on all shared TCR clone CDR3 data, where red points represent clonal CDR3s with higher expression in malignant, blue points represent clonal CDR3s with higher expression in benign, and gray points represent other clonal CDR3s **(D)** Frequency expression heatmap of TCR clonal CDR3s (p < 0.01, logFC = 2)., with significant clones clustered and normalized by standard deviation (331 malignant -associated clones and 442 benign -associated clones). **(E, F)** Sequence representation maps of significantly different malignant -associated TCR clones and benign -associated TCR clones, respectively. **(G)** Box line plots demonstrating the differences in the number of shared clonal CDR3 in BCR immune repertoires among three groups: between malignant patients, between malignant and benign patients, and between benign patients. **(H)** Volcano plots based on all shared BCR clone CDR3 data, where red points represent clonal CDR3s with higher expression in malignant, blue points represent clonal CDR3s with higher expression in benign, and gray points represent other clonal CDR3s. **(I)** Frequency expression heatmap of BCR clonal CDR3s, with significant clones (p < 0.01, logFC = 2) clustered and normalized by standard deviation (13 malignant -associated clones and 8 benign -associated clones).

Given its role as the most variable TCR clonotype and a key determinant of antigen binding, the CDR3 region was investigated to assess the clonal heterogeneity of TCR β-chain and BCR H-chain repertoires in patients with malignant and benign ovarian tumors. The count of public clonal CDR3 was analyzed across three categories: ovarian malignancy, ovarian malignant tumors, and a comparison between patients with malignant and benign ovarian tumors. The results (**Figure 3A**) demonstrated that the abundance of shared clonotypes was significantly higher in patients with benign tumors compared to those with malignant ovarian tumors, indicating greater heterogeneity in the malignant cohort (Wilcoxon test, p < 0.001). Additionally, the TCR repertoire exhibited greater variability across patient groups than the BCR repertoire, as indicated by a smaller difference in shared clonotype abundance within the BCR (**Figure 3G**) (Wilcoxon test, p<0.05). **Figure 3B** further illustrate the number of shared clonal CDR3 sequences between patients, where a deeper red coloration indicates a higher number of shared clones. These results suggest that benign tumor patients exhibit greater clonal sharing and richness, whereas malignant tumor patients have fewer shared clones and higher repertoire heterogeneity.

To systematically investigate differential clonal CDR3 expression in TCR repertoires between patients with malignant and benign ovarian tumors, all 4,741,689 TCR CDR3 clones were filtered against 117,600,502 BCR clones to exclude low-expression clones. Differential expression analysis (p < 0.01, logFC = 2) identified 331 malignant tumor-associated CDR3 clones that were preferentially expressed in malignant ovarian tumor patients, whereas 442 benign-tumor-associated CDR3 clones were preferentially expressed in patients with benign ovarian tumors (**Figure 3C**). The corresponding 773 clone CDR3 expression heat maps are shown in **Figure 3D**, of which 331 malignant-tumor-associated clones CDR3 were highly expressed in malignant patients, and 442 benign-tumor-associated clones were highly expressed in benign patients. In the BCR immune repertoire, 13 malignant-tumor-associated CDR3 clones were preferentially expressed in malignant tumor patients, while 8 benign-tumor-associated CDR3 clones were preferentially expressed in benign tumor patients (**Figures 3H, I**).

Additionally, CDR3 motifs associated with malignant (**Figure 3E**) and benign tumors (**Figure 3F**) were examined in the TCR immune repertoire. A conserved “LRGS” motif at positions 6–9 was identified among malignant-associated clones. Notably, this sequence has been reported in local immunoinflammatory diseases such as osteoarthritis (17), suggesting that it may represent a conserved inflammatory sequence pattern.

### Differential V-J gene usage in malignant and benign ovarian tumors

**Figure 4:**
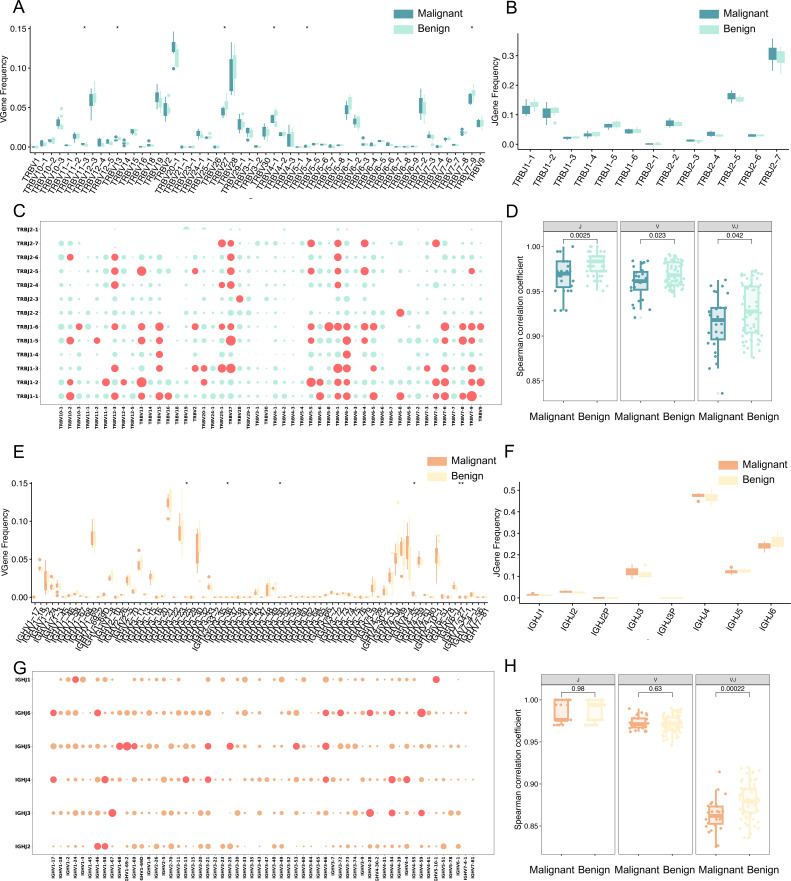
Differential analysis of TCR and BCR gene usage. **(A)** Box line plots demonstrating the difference in abundance of V genes between malignant and benign groups for TCR. **(B)** Box line plots demonstrating the difference in abundance of J genes between malignant and benign groups for TCR. **(C)** Bubble plot representing V-J paired genes for TCR, where the horizontal axis represents V genes, the vertical axis represents J genes, and each bubble represents a V-J gene combination. The size of the bubble corresponds to the -log10 p-value, with larger bubbles indicating smaller p-values and greater significance. Red bubbles indicate p < 0.05, and green bubbles indicate p > 0.05 (Wilcoxon test). **(D)** Spearman correlation coefficients of V gene, J gene, and V-J pairing between malignant and benign for TCR. The vertical axis represents the Spearman correlation coefficient values. **(E)** Box line plots demonstrating the difference in abundance of V genes between malignant and benign groups for BCR. **(F)** Box line plots demonstrating the difference in abundance of J genes between malignant and benign groups for BCR. **(G)** Bubble plot representing V-J paired genes for BCR, Red bubbles indicate p < 0.05, and orange bubbles indicate p > 0.05 (Wilcoxon test). **(H)** Spearman correlation coefficients of V gene, J gene, and V-J pairing between malignant and benign for BCR. The vertical axis represents the Spearman correlation coefficient values. Statistical significance is denoted as follows: ns (no significance, p > 0.05), *p < 0.05, **p < 0.01, ***p < 0.001 (Wilcoxon test).

To analyze the distribution of TCRβ V and J genes, the frequencies of V gene and J gene fragments were calculated for each sample. The Wilcoxon test (p < 0.05, uncorrected) identified six V gene segments with significantly different usage between patient groups (**Figure 4A**). Specifically, TRBV11-3, TRBV19, TRBV24-1, TRBV7-7, and TRBV5–4 were significantly more frequent in malignant patients, whereas TRBV27 was more frequent in benign patients. These findings are consistent with previous reports of similar gene fragment enrichment in diseases such as breast cancer (18), chronic myeloid leukemia (19), COVID-19 (20), and colorectal cancer (21), suggesting that the expansion of these TCRβ V gene clones may result from tumor antigen stimulation. The J gene fragments, however, did not exhibit significant differences (**Figure 4B**). In addition, the usage patterns of TCRβ V-J pairings in malignant and benign patient populations were compared, and the red bubbles in **Figure 4C** indicate a significant difference in 75 pairs of dominant V-J paired genes in the two patient populations (p<0.05, Wilcoxon test, uncorrected). Using 48 V gene segments, 13 J gene segments, and 624 V-J gene combinations, the Spearman correlation coefficient between patients with malignant tumors and the benign tumors was calculated (**Figure 4D**). Spearman correlation coefficient analysis of V-J gene combinations showed significantly lower correlation among malignant patients compared to benign patients, indicating high heterogeneity in TCR gene usage among malignant cases.

For the BCR H chain, Wilcoxon analysis (p < 0.05, uncorrected) identified seven V gene segments from 58 V gene with significantly different usage between malignant and benign groups (**Figure 4E**), including IGHV3-25, IGHV3-30, IGHV3-47, IGHV3-60, IGHV3-62, IGHV3-64, and IGHV4-55. The J gene fragments, however, did not exhibit significant differences (**Figure 4F**). Notably, IGHV3–30 and IGHV3–64 have been implicated in chronic lymphocytic leukemia (22) and head and neck squamous cell carcinoma (23). The analysis also revealed 40 significantly different dominant V-J pairs from 348 V-J genes, as shown by the red bubble in **Figure 4G** (p<0.05, Wilcoxon test, uncorrected), reinforcing the hypothesis that these amplified clones result from tumor antigen-driven selection. The Spearman correlation coefficient between patients with malignant and benign tumors was calculated for 58 V gene segments, 6 J gene segments, and 348 V-J gene combinations (**Figure 4H**), and the higher the value of Spearman correlation coefficient, the more correlated the patients were, and the correlation between patients with malignant tumors was significantly lower than that of patients with benign tumors. Compared with TCR, the V-J gene specificity of BCR is much lower, suggesting that the V-J paired gene profile of BCR is highly heterogeneous in patients with malignant tumors.

### Early machine learning-based screening model for distinguishing benign and malignant ovarian tumors

**Figure 5:**
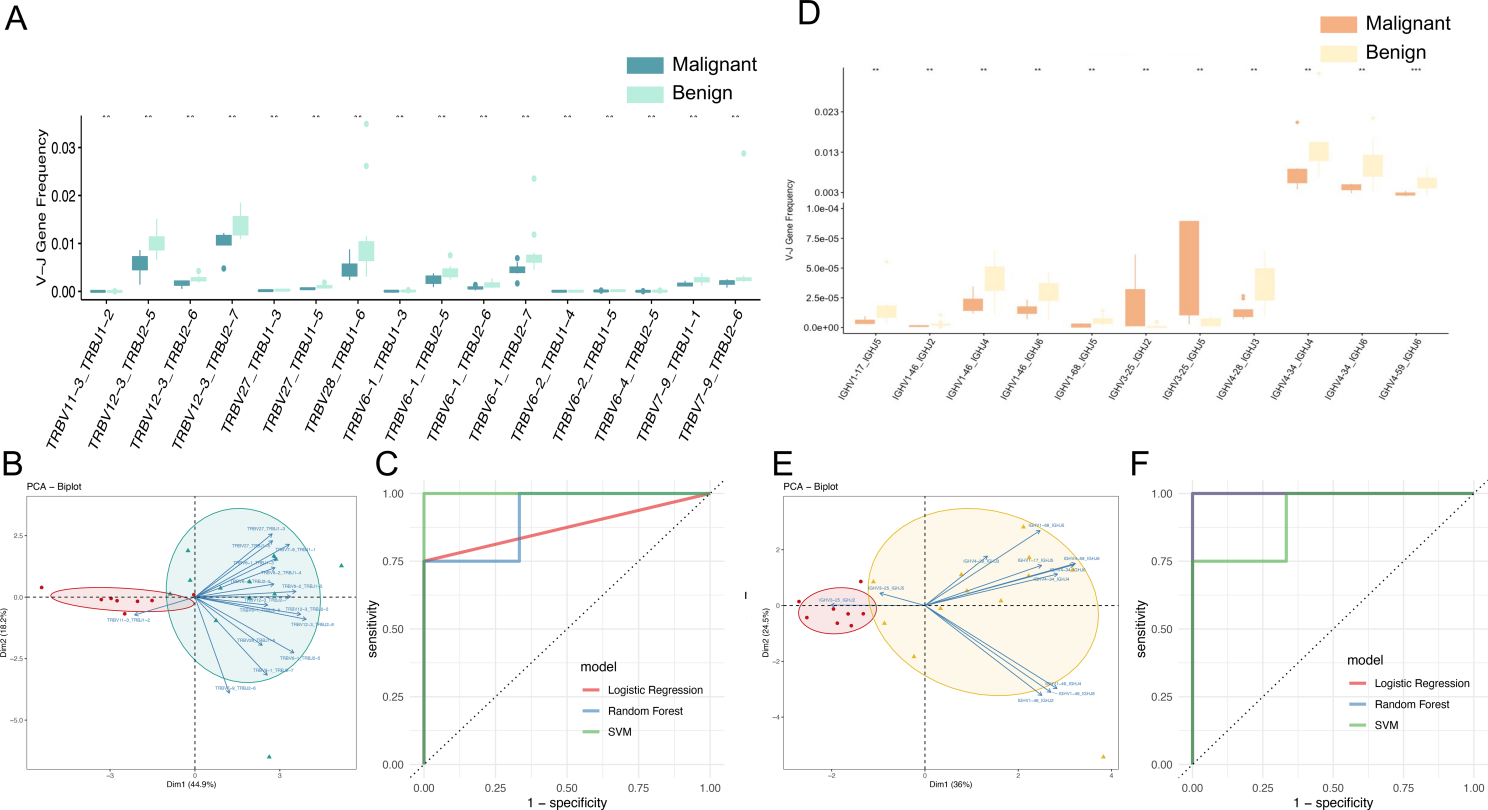
TCR and BCR V-J genes as potential biomarkers for diagnosing benign and malignant groups. **(A)** Box line plot demonstrating the significant abundance difference of TCR V-J genes (**p < 0.01; Wilcoxon test) between the malignant and benign groups. **(B)** Principal component analysis (PCA) based on the usage of TCR V-J paired genes, with vectors representing characterized V-J genes, red dots representing malignant patients, and green dots representing benign patients. **(C)** ROC curves and AUC values of the model prediction results for early diagnosis of benign and malignant ovarian tumors using TCR V-J genes by Support Vector Machine (SVM), Random Forest (RF) and Logistic Regression (LR) models. **(D)** Box line plot demonstrating the significant abundance difference of BCR V-J genes (**p < 0.01; Wilcoxon test) between the malignant and benign groups. **(E)** Principal component analysis (PCA) based on the usage of BCR V-J paired genes, with vectors representing characterized V-J genes, red dots representing malignant patients, and yellow dots representing benign patients. **(F)** ROC curves and AUC values of the model prediction results for early diagnosis of benign and malignant ovarian tumors using BCR V-J genes.

The gene rearrangement of TCR/BCR gene rearrangement in peripheral blood is crucial for tumor immune responses. This study investigated whether TCR/BCR V-J pairing characteristics in peripheral blood could serve as diagnostic biomarkers for ovarian tumors by analyzing data from eight patients with malignant ovarian tumors and twelve patients with benign ovarian tumors. The results showed that there was a significant difference in the pairing of TCRβ V-J in peripheral blood between malignant and benign patients, indicating that both malignant and benign patients had a unique TCR/BCR immune repertoire. Further analysis identified 75 V-J pairs with differential abundance (p<0.05, uncorrected), including 16 highly significant pairs (p<0.01), though these exploratory findings remain uncorrected for multiple testing due to sample size limitations and require validation in larger cohorts (**Figure 5A**). Notably, TRBV11-3_TRBJ1–2 was more abundant in malignant cases, suggesting specific amplification. The 16 pairs of V-J genes are TRBV11-3_TRBJ1-2, TRBV12-3_TRBJ2-5, TRBV12-3_TRBJ2-6, TRBV12-3_TRBJ2-7, TRBV27_TRBJ1-3, TRBV28_TRBJ1-6, TRBV6-1_TRBJ1-3, TRBV6-1_TRBJ2-5, TRBV6-1_TRBJ2-6, TRBV6-1_ TRBJ2-7, TRBV6-2_TRBJ1-4, TRBV6-2_TRBJ1-5, TRBV6-4_TRBJ2-5, TRBV7-9_TRBJ1-1, TRBV7-9_TRBJ2-6. Based on the 16 pairs of V-J genes with significant differences as characteristics, the PCA dimensionality reduction treatment was carried out, and obvious clustering between patients with ovarian malignant tumors and patients with benign ovarian tumors was observed in the PCA diagram (**Figure 5B**), and it was preliminarily indicated that these differential V-J genes can effectively distinguish between malignant patients and benign patients, which will help identify potential biomarkers, so as to provide new biomarkers and therapeutic targets for the early diagnosis and treatment of ovarian tumors.

In order to establish a robust biomarker model for early diagnosis of benign and malignant tumors of peripheral blood and ovaries, the following steps were carried out: (1) Data preprocessing: cleaning the V-J gene data in peripheral blood samples, including removing outliers and missing values, and standardizing the frequency data (mean-centering without scaling) of V-J genes to ensure scale consistency of different genes. (2) Feature selection: 16 pairs of V-J genes based on significant differences were used as input for training features. (3) Data partitioning: The data set is divided into training set and test set, and the samples are randomly divided into 70% of the training data set and 30% of the test data set (i.e., 14 training samples and 6 test samples) according to the category ratio. (4) Model construction: select machine learning algorithms Support Vector Machine (SVM), Random Forest (RF) and Logistic Regression (LR). The model was trained using the selected machine learning algorithm on the training dataset, and the expression data of 16 pairs of V-J genes were used as input variables, and the sample labels (patients with benign or malignant ovarian tumors) were used as output variables. In the test data set, the AUC of different models of patients with benign and malignant ovarian tumors were logistic regression: 0.875, random forest: 0.917, and SVM: 1, respectively (**Figure 5C**). The mean AUC for the three models was 0.93. These results suggest that the use of TCRβV-J gene in peripheral blood has a strong discriminative ability and can effectively identify patients with benign and malignant ovarian tumors, which is a promising biomarker for early diagnosis.

Among the 40 pairs of BCR V-J genes exhibiting differential abundance between malignant and benign tumors patients, 11 pairs demonstrated statistically significant differences (p < 0.01), though these exploratory findings remain uncorrected for multiple testing due to sample size limitations and require validation in larger cohorts, with IGHV3-25_IGHJ5 and IGHV3-25_IGHJ2 being the V-J pairs more abundant in malignant tumors patients. The 11 significantly different V-J gene pairs (**Figure 5D**) included IGHV1-17_IGHJ5, IGHV1-46_IGHJ2, IGHV1-46_IGHJ4, IGHV1-46_IGHJ6, IGHV1-68_IGHJ5, IGHV3-25_IGHJ2, IGHV3-25_IGHJ5, IGHV4-28_IGHJ3, IGHV4-34_IGHJ4, IGHV4-34_IGHJ6, and IGHV4-59_IGHJ6. The application of Principal Component Analysis (PCA) to the 11 pairs of V-J gene pairs in question yielded a clear distinction between malignant and benign cases, as illustrated in **Figure 5E**. Utilizing the same data processing pipeline and machine learning models, predictive modeling was conducted based on BCR V-J gene pairs. The area under the curve (AUC) for distinguishing malignant from benign cases was 1.0 for Logistic Regression, 1.0 for Random Forest, and 0.875 for SVM (**Figure 5F**), with an average AUC of 0.958 across models. This finding indicates that these differential V-J genes can effectively distinguish between patients with benign and malignant tumors, which is beneficial for identifying new biomarkers and therapeutic targets for the early diagnosis and treatment of ovarian tumors.

### Distinct spatial distribution patterns of TCR and BCR between benign and malignant tumors

**Figure 6:**
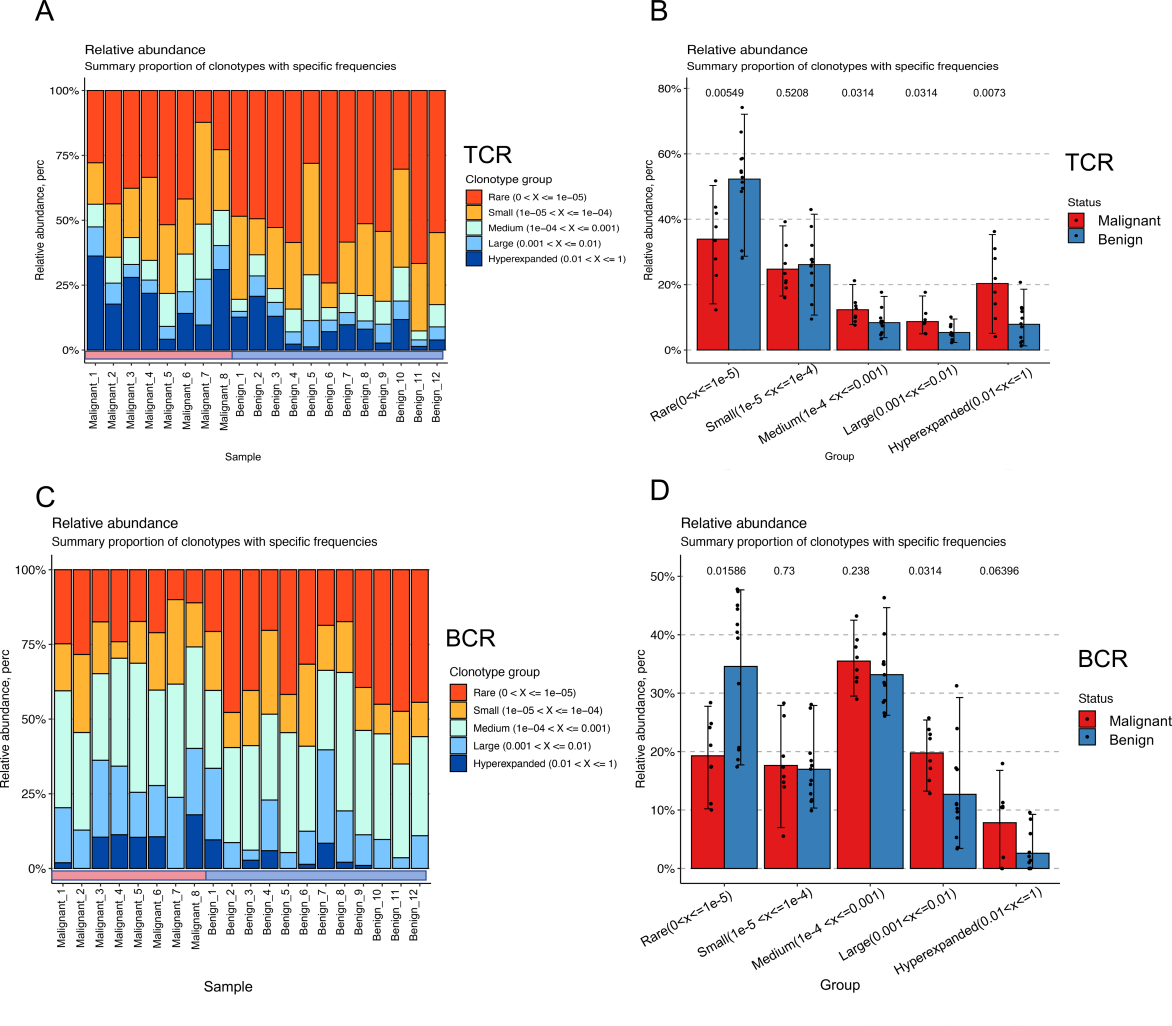
Spatial distribution shifts in TCR and BCR between benign and malignant groups. **(A)** Bar-pair stacked plot demonstrating the spatial distribution of TCR clones in all samples. Clones are categorized into five groups based on their frequency: Rare (0 < x < 1e-05, red), Small (1e-05 < x < 1e-04, yellow), Medium (1e-04 < x < 1e-03, green), Large (1e-03 < x < 1e-02, light blue), and Hyper-expanded (0.01 < x < 1, blue). The horizontal axis represents individual samples, with the first eight samples corresponding to the malignant group and the last eight to the benign group. **(B)** Box plot showing the variation in clonal spatial steady-state differences between the malignant (red) and benign (blue) groups for TCR (Wilcoxon test). **(C)** Bar-pair stacked plot demonstrating the spatial distribution of BCR clones in all samples. Clones are categorized into the same five frequency-based groups as in **(A)**. The horizontal axis represents individual samples, with the first eight samples corresponding to the malignant group and the last eight to the benign group. **(D)** Box plot showing the variation in clonal spatial steady-state differences between the malignant (red) and benign (blue) groups for BCR (Wilcoxon test).

TCR and BCR in peripheral blood represent two distinct types of immune receptors. The findings above indicated that the distribution patterns of TCR and BCR in benign and malignant tumor populations are not entirely consistent. Therefore, we conducted a more profound investigation to elucidate the spatial distribution with respect to BCR and TCR between benign and malignant tumors who are more variable. To investigate the clonal distribution of TCR and BCR repertoires, we analyzed the proportion of clones within specific size ranges in the pooled samples. In TCR repertoires (**Figure 6A**), the rare clone group (Rare: 0 < x < 1e-05) constituted the largest proportion of the total clonal space, followed by the small clone group (Small: 1e-05 < x < 1e-04). In contrast, BCR repertoires (**Figure 6C**) were predominantly composed of clones from the medium clone group (Medium: 1e-05 < x < 1e-04) and the rare clone group. When comparing malignant and benign ovarian tumor populations, significant differences in clonal distribution were observed. In TCR repertoires (**Figure 6B**), the malignant population exhibited a higher proportion of clones in the medium clone group, large clone group (Large: 1e-03 < x < 1e-02), and hyper-expanded clone group (Hyper-expanded: 0.01 < x < 1) compared to the benign population (p < 0.05). Similarly, in BCR repertoires (**Figure 6D**), the malignant population showed a marked increase in expanded clones, particularly within the large and hyper-expanded clone groups (p < 0.05). Further analysis revealed that the expanded clones in malignant populations primarily originated from rare clone groups. Our analysis revealed distinct clonal distribution patterns between TCR and BCR repertoires in malignant versus benign ovarian tumors. The malignant tumor populations showed significantly higher proportions of expanded clones (medium, large and hyper-expanded groups) compared to benign populations, with more pronounced differences observed in TCR repertoires (p=0.00549) than in BCR (p=0.015). Meanwhile, rare clone groups in benign tumors were substantially more prevalent representation in malignant tumors, particularly in TCR repertoires. These findings demonstrate characteristic differences in immune repertoire architectures between disease states, with TCR repertoires exhibiting greater inter-group variation than BCR repertoires. These findings provide valuable insights into the immune mechanisms underlying ovarian tumor progression and may inform the development of immune-based diagnostic and therapeutic strategies.

## Discussion

In this study, we investigated whether immune receptor sequencing could serve as a reflection of a person’s ovarian benign or malignant tumor status, based on autoreactivity shaping the immune system’s collection of adaptive immune receptors. The immune repertoires analysis was implemented in order to assess the immune responses of 20 individuals with well-defined datasets of two disease tumor immunological states leveraging both BCR and TCR to achieve. Exposure to early ovarian tumor antigens may lead to rapid expansion of cancer-associated T cells and B cells, resulting in a detectable TCR/BCR repertoire signal in circulating leukocytes. Faced with highly diverse sequence repertoires containing tens to hundreds of thousands of different sequences, the analysis reveals differences in the state of the immune repertoire of benign malignant tumor populations and prioritizes the selection of disease-specific sequences and V/V-j genes for predicting of ovarian tumor. By deepening the exploration of the TCR/BCR immune repertoires, the process of the occurrence and development of immune complexes in the process of tumor progression from benign to malignant has become clearer, and this change reveals important changes in the immune response, which is conducive to the understanding of tumor deterioration at the immune level.

The observed reduction in TCR and BCR repertoire diversity, balance, and richness in malignant ovarian tumors aligns with previous findings in other cancer types, suggesting a conserved mechanism of immune evasion or exhaustion in malignant states. The decreased diversity may reflect the clonal expansion of tumor-specific T and B cells, which dominate the repertoire and reduce its overall heterogeneity. This phenomenon is further supported by the lower abundance of shared clonal CDR3 sequences in malignant cases, indicating a more fragmented and less coordinated immune response. The age-related decline in repertoire diversity adds another layer of complexity, potentially reflecting immunosenescence or cumulative antigen exposure over time. These findings underscore the dynamic interplay between tumor evolution and immune adaptation, highlighting the need for longitudinal studies to track repertoire changes throughout disease progression.

The identification of differential V genes, V-J gene pairs, and CDR3 sequences provides a foundation for developing immune-based biomarkers for ovarian tumors. Notably, the TCR repertoire exhibited more pronounced changes than the BCR repertoire, with 773 differentially expressed CDR3s compared to only 21 in BCR, suggesting that TCR may play a more central role in anti-tumor immunity and could serve as a more sensitive indicator of malignant transformation. The machine learning models, which are the BCR-based and the TCR-based model, demonstrates the potential of immune repertoire features for early tumor detection. However, the stability and generalizability of these models require further validation in larger, multi-center cohorts. Future studies should also explore the functional relevance of the identified differential sequences and their potential as therapeutic targets.

This study provides novel insights into the differences in the TCR and BCR repertoires between benign and malignant ovarian tumors, highlighting a reduction in immune repertoire diversity, balance, and richness in malignant cases. Furthermore, our machine learning model, based on immune repertoire features, demonstrated high predictive performance, indicating the potential of immune-based approaches for early tumor screening.

However, this study remains exploratory, with several limitations that warrant further investigation. The lack of a precise numerical threshold to differentiate benign from malignant tumors limits its immediate clinical applicability. Additionally, the observed correlation between age and immune repertoire diversity requires validation in a more diverse cohort with a broader age range. While our machine learning model achieved promising results, its stability across different models remains a challenge, emphasizing the need for larger datasets to enhance robustness and reproducibility. Despite these challenges, our findings reinforce the role of immune repertoire analysis in understanding ovarian tumor progression and highlight the potential of prospectively collected immune data in biomarker discovery. Future research with expanded sample sizes and refined methodologies could pave the way for immune-based early detection strategies in ovarian cancer.

## Conclusions

In this study, we conducted a comprehensive analysis of the TCR and BCR immune repertoires in patients with benign and malignant ovarian tumors, yielding several significant findings: (1) Patients with malignant ovarian tumors exhibited distinct immune repertoire characteristics, including reduced in balance, richness, and diversity clonotype abundance in both TCR and BCR repertoires compared to benign cases. (2) Furthermore, we observed an age-related decline in the diversity of both TCR and BCR immune repertoires were observed. (3) The investigation of shared clonal CDR3 sequences indicated that malignant tumor patients exhibited diminished abundance and elevated heterogeneity, as demonstrated by diminished Spearman correlation coefficients based on TCR, J genes, V-J genes, and BCR V-J genes in comparison to benign cases. This finding underscores the increased immunological heterogeneity in malignant ovarian tumors. (4) Through differential gene expression analysis, we identified substantial variations between the two groups: 6 differential V genes, 75 differential V-J genes, and 773 differentially expressed CDR3 in the TCR repertoire, along with 7 differential V genes, 40 differential V-J genes, and 21 differentially expressed CDR3 in the BCR repertoire. (5) Leveraging these differential features, we developed machine learning models for early tumor screening. The TCR-based model, incorporating 16 significant V-J gene pairs (p<0.01), achieved a Mean-AUC of 0.93, while the BCR-based model, utilizing 11 significant V-J gene pairs, demonstrated superior performance with a Mean-AUC of 0.958. These models show promising potential for improving early detection rates and treatment outcomes in ovarian tumors. (6) Comparative analysis of TCR and BCR spatial distribution in peripheral blood demonstrated significantly greater differences in TCR patterns between benign and malignant states, with 773 differential CDR3s identified in TCR versus only 21 in BCR. This observation, coupled with the more prominent differential V-J gene expression in TCR, suggests that TCR may play a more significant role in the immune response to ovarian tumor progression. These findings suggest that TCR could serve as a valuable biomarker for ovarian tumor characterization and further investigation in studies.

The distinct patterns observed in TCR and BCR repertoires may reflect fundamental differences in their respective roles in anti-tumor immunity, with TCR potentially serving as a more sensitive indicator of malignant transformation. This study provides valuable insights into the immunological landscape of ovarian tumors and establishes a foundation for the development of novel diagnostic and prognostic tools in ovarian cancer management.

## Data Availability

The data presented in the study are deposited in the OMIX repository, China National Center for Bioinformation / Beijing Institute of Genomics, Chinese Academy of Sciences (https://ngdc.cncb.ac.cn/search/specific?db=bioproject&q=PRJCA043993) (accessed [2025-12-31]) (24, 25).
