# Supplementary material for: TCR and BCR repertoire analysis reveals distinct signatures between benign and malignant ovarian tumors
Source: Front Oncol. 2025 Aug 11;15:1630707. doi: 10.3389/fonc.2025.1630707 (PMC12375485; doi:10.3389/fonc.2025.1630707)

Figure S1

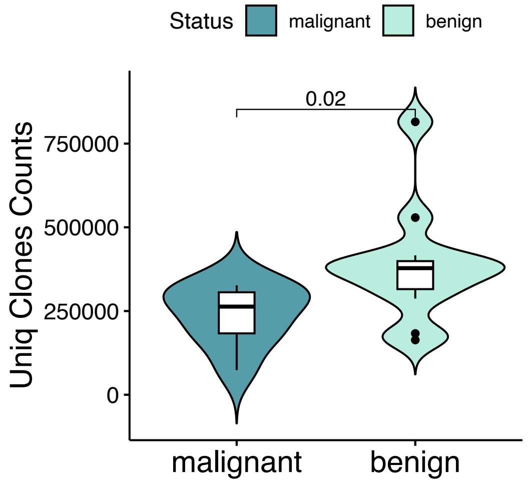

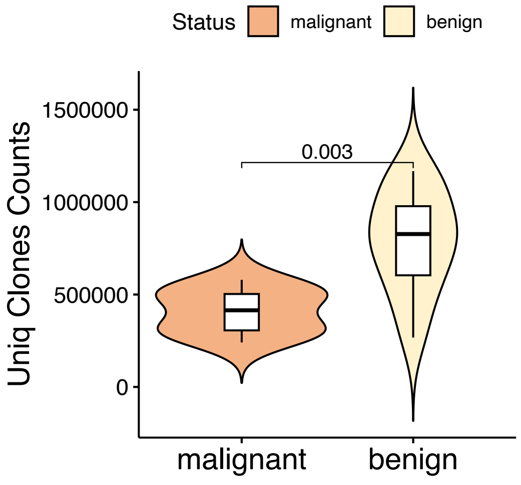

Figure S2

TCR Gini index- Gravida/Para/Abortus

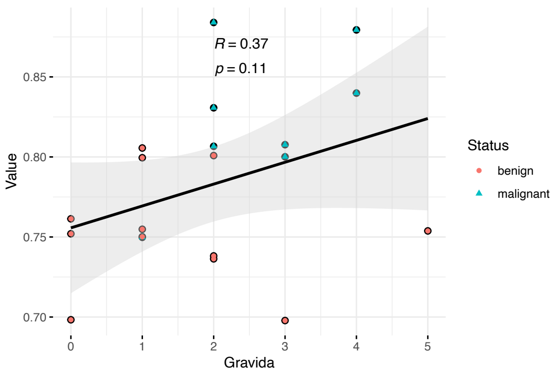

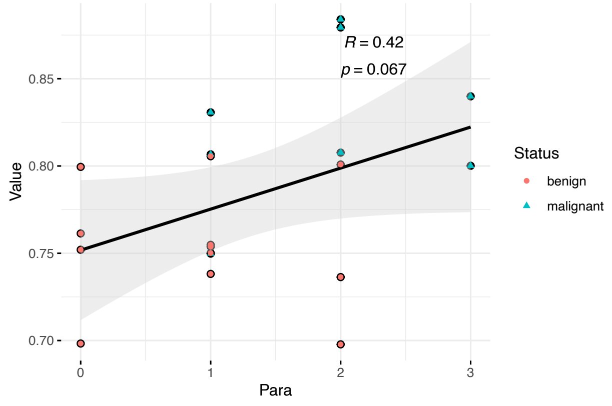

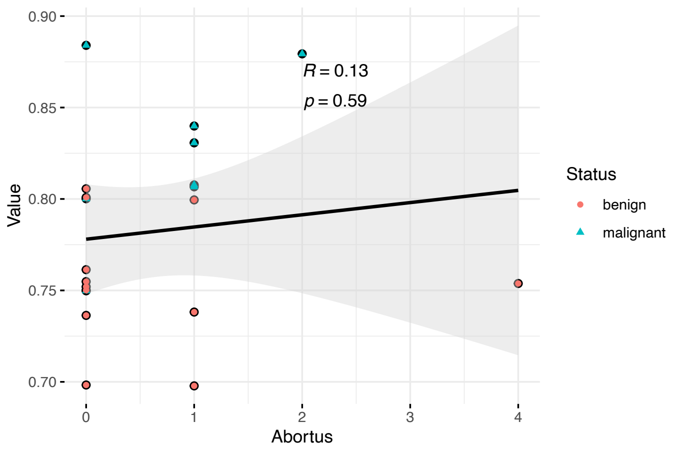

Figure S3

BCR Gini index- Gravida/Para/Abortus

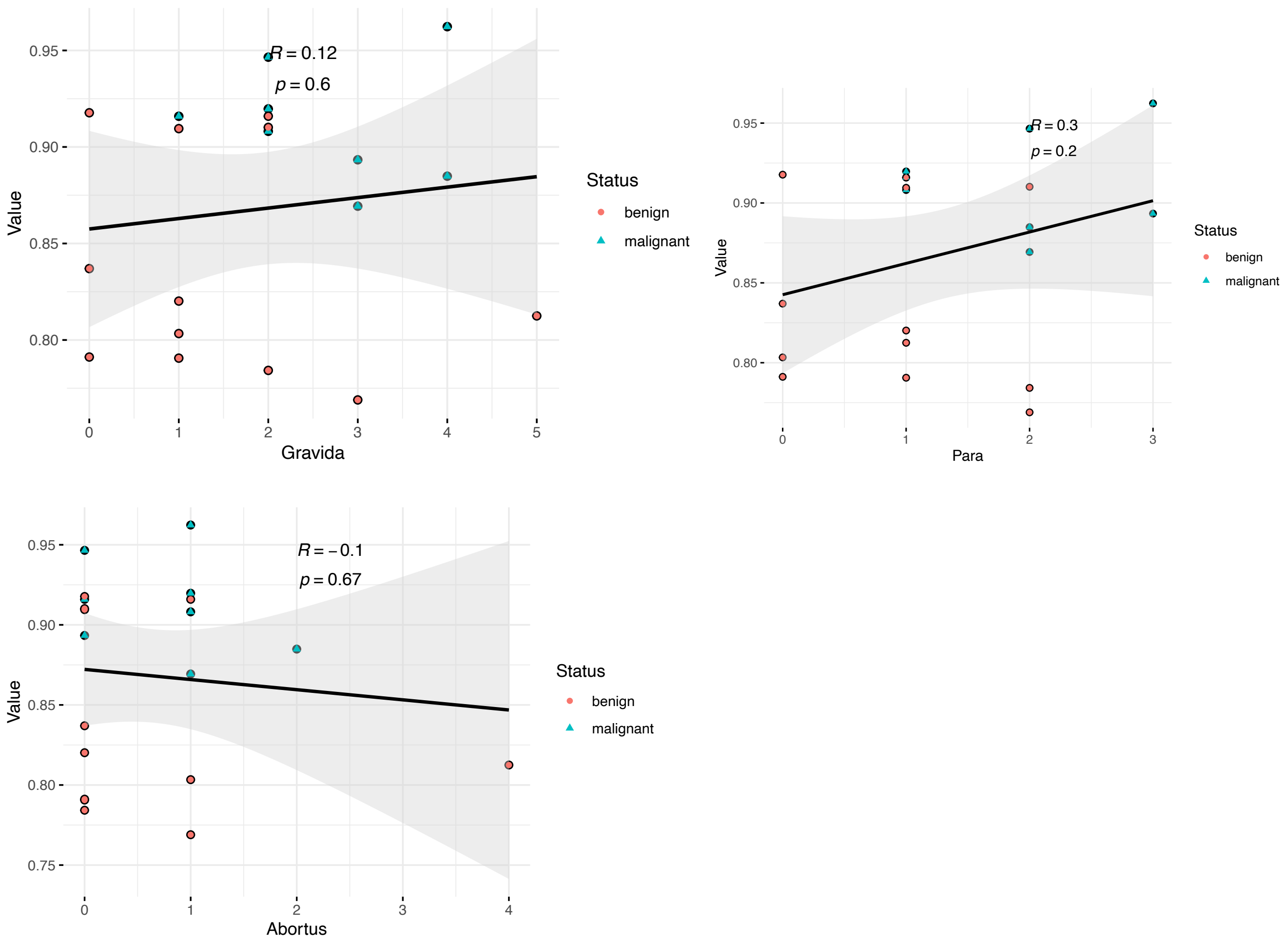

Supplement: Supplementary file 1 [file DataSheet1.pdf]
